# Psychological well-being of substance use patient: Role of religious therapy as the treatment

**DOI:** 10.12669/pjms.35.5.561

**Published:** 2019

**Authors:** Zaqia Bano, Iram Naz, Naeem Leghari, Ishtiaq Ahmed

**Affiliations:** 1 Dr. Zaqia Bano, Department of Psychology, University of Gujrat, Pakistan; 2 Iram Naz, Department of Psychology, University of Gujrat, Pakistan; 3 Dr. Naeem Ullah Leghari, Head, Psychiatry Ward, Nishter Hospital, Multan, Pakistan; 4 Dr. Ishtiaq Ahmed, Al-Nafees Medical College, Isra University, Islamabad Campus, Pakistan

**Keywords:** Drug addicts, Patient, Pharmacotherapy, Psychological well-being, Religious therapy, Substance use, Therapy

## Abstract

**Objective:**

To evaluate the psychological well-being of substance use patients in comparison of combined religious therapy and pharmacotherapy effects with that of pharmacotherapy effects alone and also to assess the psychological well-being of drug addicts in comparison of demographics characteristics.

**Methods:**

This experimental study was conducted at Department of Psychology, University of Gujrat from July 5^th^ 2016 to July 25^th^ 2017. A sample of 140 drug addicted patients was taken from different hospital. The pre and post-test of experimental and control group was done. Experimental group received standard pharmacotherapy along with religious therapy while patients in the control group only induced standard pharmacotherapy. The effectiveness of therapy was judged on their psychological well-being using California Psychological Inventory Well-being sub-scale..

**Results:**

The results of the study confirmed that there was significant difference in the psychological well-being of control and experimental group (p-value <0.01). After the treatment a difference exists in the means of control and experimental (16.24 and 26.44 respectively) groups. An increase in the psychological well-being of those having religious therapy comparing to those not having religious therapy was observed. Further, comparing the demographic variables the means indicated that treatment affected all age groups, marital status and education level equally. Whereas, in the rural area (mean, 27.04) psychological well-being was better than the urban (mean=26.11) and with the income levels of 21,000 to 30,000 (mean, 27.57) there was more improvement as compared with other income levels (mean, 26.35 and mean, 26.03).

**Conclusion:**

The religious therapy had a significant therapeutic effect on psychological well-being of the substance use patients and it is equally effective for all age group, marital status and educational level.

## INTRODUCTION

The diagnostic criteria of the Diagnostic and Statistical Manual of Mental Disorders^1^ well-defined substance used disorders as a group of psychosomatic symptoms that exist with different cognitive and behaviour related problems. The global prevalence of substance used disorders is not only common but its rate is increasing considerably.^2^ The World Drug Report^3^ has articulated that a quarter of billion people used drugs at least once a time in 2015. Furthermore, it was also testified that 29.5 million adult segment of the people were having substance used disorders internationally. Moreover, 3.3 million of the population died due to alcohol use every year with 15.3 million people fulfils the substance use disorder criteria. Finally, there were 255 million people used illegal drugs with 12 million injectable uses of drugs. If we talk about Pakistan, the substance use was also alarming. There were 6.7% of the persons was in use of illegal drugs and about 4.3% were the drug users. The use of drugs leads to restriction of everyday life activities. The substance use linked with number of negative effects that may include decline in physical ability, social and psychological relapses.

The National Institute of Drug Abuse^4^ reported that use of substance can be a reverting and a persistent illness for the brain acquired through the use or desire to use the drugs without paying attention or thinking about the side effects of it. Substance use disorder had feelings that resulted in lack of control and an inability. These individual were in the great requirement of treatment. Furthermore, this large and regular use of drugs also enhanced the occurrence of other psychiatric disorders. Besides, stigmatizing the drug use restricting the social support of addicts ultimately leads towards depression and other psychiatric illness.^5^

A number of clinicians and specialists were putting efforts to articulate effective treatment plan to make the retrieval of drug abusers as easy and valuable.^6^ It is important to note that the intervention commonly used for drug users are in systematic pattern, which started with the first step of detoxification though medicine, then psychological testing and assessment which finalized with therapies that can be performed in or outside of the rehabilitation center. Beside, others religious therapy is a significant treatment method which is very supportive and showed positive outcomes.^7^ Furthermore, it is worth to note that the religious beliefs are not a contributor in drug use but work as an inhibiting power.^8^ According to religious perspective, person who seek help from God to cope with their illness and distress have less anxious and depressive feelings. It is because of the religious beliefs that reinforces individuals’ in improving their psychological well-being.^9^

The meditation is a widely experienced and a central practice that can discover or develop the highest psychological or religious levels. The meditation therapy is essentially accompanying a good sense of oneself, active method of mind, relaxation and positive foundation of energy, which add up a person thinking to have better understanding about the psychological problem and to find better explanations to it. It has been confirmed that meditation can easily terminate anxiety, enhance psychological well-being, empowers human will power, promote satisfaction with self and raised confidence.^10^ The 12-step program highlights the higher power of religious believe system and individual turned his/her intention back toward God. Generally, this traditional 12-step program is for alcoholics but proved to be more effective and useful intervention for drug addicts also. In the nineteenth century a number of treatment organization used alcoholics anonymous (AA) approach for using public commitment and confession a mutual aid concept.^2^ It is commonly observed that people have a strong commitment to religious activities, probably, they develop stronger beliefs in 12-steps program as compared to others, because the program is a concept which try to help the individual to develop strong beliefs about Allah and it will help to encourage will power in a person.^10^

According to National Centre on Addiction and Substance Abuse the individuals that were involved in smoking, use alcohol and marijuana and also indulged to take part in the religious practices for a one week, shows a significant reduction in their addictive behavior.^11^ It was also confirmed that religion is a protected factor for individuals not to indulge into addictive behaviour and could be used as a preventive measure for stopping people from addictions and could be used as cure to drug use.^12^

In Pakistan, the substance use creating devastating problems and most importantly the issue is accumulating by the passage of time. About 6% of the Pakistani population was using drugs as reported by United Nations Office on Drug and Crime.^13^ The consequences may include health concerns, prosperity loss, disrespect, emotional issues and stigma. Further, the substance users were perceived by the general people as dangerous, deceitful, undependable, impulsive and with many moral disbeliefs and these people blame the drug users as being responsible for their current situation.^5^ Studies have showed that the weak religious beliefs also become an important factor to enhance the substance use in our country.^14^

The current research considers the religious therapy as treatment for enhancing the psychological well-being of substance use patients. The study finds out the effectiveness or appropriateness of religious therapy as the intervention to handle the psychological well-being of drug addicts. Pakistan is a religious society and systematic way of religious techniques as treatment will provide a new basis of intervention. The study was conducted with an objective to evaluate the psychological well-being of substance use patients in contrast of both religious therapy and pharmacotherapy effects with that of pharmacotherapy affects alone and to assess the psychological well-being of drug addicts in comparison of demographics characteristics.

## METHODS

This experimental study was conducted at Department of Psychology, University of Gujrat from July 5^th^ 2016 to July 25^th^ 2017. From private hospital, the sample of 140 diagnosed male patients of substance use disorder was recruited. Only those patients were taken whose stay in the hospital was at least three or more than three months. The patients with co-occurrence of any other health problem or psychiatric disorder were excluded from the study.

The study used Pre and Post-test with control group and experimental groups. Experimental group consisted of patients who received the religious therapy and medication whereas; the control group received only medication. The psychological well-being was measured by using California Psychological Inventory (well-being scale) by Ghough developed in 1987.^15^ Median internal consistency of the scale was 0.76. The demographic like age, marital status, residential type, education and income of patients were also studied.

Initially, research proposal was discussed with clinical psychologist and psychiatrist to review the ethical concerns and study design. Then approval from the establishment of hospital in Multan and Gujrat was taken for data collection. Before starting the intervention, the psychological well-being of the diagnosed substance use patients was assessed. One hundred forty patients were matched pair on sample characteristics and then randomly assigned to experimental and control group. Religious therapy is one of the widely used therapies for drug addiction treatment. According to Kazdin^16^ the people who are devoted religiously and engross in religious coping behaviors like praying, usually have better well-being and life satisfaction. Ajmal^17^ said prayer, commemoration of God, is an act of worship which get someone closer to higher power and this closeness and intimation leads to divine illumination. Reading therapy is a method to read problem related fact as proposed by therapist, instead of direct meeting, in order to gain intuition about the problem. Rizvi^18^ prescribed that the most effective therapeutic technique, selective reading, in which one have to follow the instruction carefully and act accordingly. Another widely used technique is a meditation technique, it is a matter of disciplinary practice for exploration of utmost psychological or religious altitudes.^19^ It also identified as self-regulatory approach for both religious and spiritual behaviors and resulted in optimistic health outcomes like dropping blood pressure as well as lessening the pain and anxiety. Religion is a supportive nurturing faithfulness. Hence, 12 steps program, an influential tool to treat substance abused, also associated with alcoholics anonymous (AA) approach. It lowers the level and magnitude of drug including licit drug and alcohol usage.^20^

In the therapy sessions the following religious therapy techniques were used as intervention i.e., reading therapy, prayer, mediation and 12 step programs. In sessions patients were asked to pray with more attentiveness and devotion. They were initiated to ask support from Allah and to perform meditation regularly before going to sleep at night and early morning. At earlier the patient felt problem in meditation, but with continuing effort they felt comfortable. The patient was also asked to follow 12-steps daily and reading material was also provided for improving religious concepts. For the uneducated participants the reading therapy was provided in a form of lecture. The group therapy was employed to address the common problems of the patients. If the patient rejected to participate in group therapy, then the individual therapy was used. During treatment at least 10 sessions were given to each patient. After the completion of therapy in the last session the post test was conducted to measure the psychological well-being again.

### Data Analysis

The data was analysed using paired sample t-test. It is also called as dependent sample t-test that is used to foresee whether there is difference in the means of two sets of observations. Further in paired sample t-test the participant is measure twice as in pre and posttest setting thus, provide pairs of observations. Statistical analysis was evaluated through SPSS version 16.

## RESULTS

A total of 140 patients were studied and equally divided in experimental (N=70) and control (70) groups. The data collected from the respondents to check religious therapy effect on the psychological well-being of drug addicts were shown.

The results of the comparison of psychological-well-being before and after treatment of experimental and control group are shown in Table-I. There are significant mean differences among drug addicted patients’ pre and post-test scores of the experimental group on psychological well-being scores (17.51 vs 26.44 respectively). This shows that the treatment has an effect on experimental group. In control group, there was a difference in the pre and posttests existed but on verifying the means there was slight high mean scores in pretest treatment as compared to posttest (17.26 vs. 16.24 respectively). This slight difference indicated that there was no intervention employed to the control group.

**Table I:** Comparison of different statistical scores of experimental and control group of drug addicts on psychological well-being in pre and posttests (N=140)

Groups	Pre-test		Post-test		t	p
	Mean	SD	Mean	SD		
Control Group (N=70)	17.26	2.36	16.24	2.34	2.56	0.01
Experimental Group (N=70)	17.51	2.20	26.44	2.68	-21.55	0.00

p<0.05 is statistically significant.

The results of psychological well-being with reference of demographic variables of age, marital status, education, residential type and income of drug addicts are shown in Table-II. The results indicated that P-value was statistically significant for all the demographic variables which means that the treatment have a significant effect on both groups on psychology well-being scores. If we compare the demographic variables the means indicated that treatment affected both age groups equally as the means of posttest is almost same. Further, as previous pattern, the marital status and education means were also almost same in the posttest. Moreover, in case of residential types rural psychological well-being was better than the urban (27.04 vs. 26.11 respectively). Finally, on the income levels of 21,000 to 30,000 shows more improvement in psychological well-being as compare to below 11,000 to 21,000 and less than 11,000 incomes levels (27.57, vs. 26.35 & 26.03 respectively).

**Table II:** Statistical comparison of demographic variables and psychological well-being in pre-posttests of experiment group (n=70).

Variables	Groups	N	Pretest		Posttest		t	p

			Mean	SD	Mean	SD		
Age	Below 30 years	41	17.37	5.19	26.51	7.16	-16.67	0.00
	Above 30 years	29	17.72	4.42	26.34	7.45	-13.47	0.00
Marital Status	Unmarried	41	17.17	5.25	26.63	7.09	-17.25	0.00
	Married	29	18.00	4.00	26.17	7.43	-13.02	0.00
Education	Matric and Below	46	17.43	5.27	26.43	7.76	-16.91	0.00
	Above Matric	24	17.67	4.14	26.46	6.35	-13.30	0.00
Residential Type	Urban	45	17.62	4.20	26.11	5.11	-16.45	0.00
	Rural	25	17.32	2.17	27.04	2.68	-14.07	0.00
Income	Less than 10,000	33	17.21	5.61	26.03	6.78	-14.39	0.00
	11,000 to 20,000	23	17.39	3.98	26.35	5.96	-13.62	0.00
	21,000 to 30,000	14	18.43	3.96	27.57	9.49	-9.33	0.00

## DISCUSSIONS

The obsession for drug is dangerous not only for the patients but also for their family members, friends, employer and society highlighted and this issue becomes a serious concern for health in country. Therefore, various treatment methods, techniques and designs were evaluated as a part of clinical efforts. Further, the treatment of substance use is problematic, either because of the compulsive nature of the substance use, or because numerous additional psychological dysfunctions that are most probable. The psychological problems may be depressive symptoms, anxiousness and a number of disturbances in their societal and work-related functioning.

Recent research has suggested that the spiritual practices can easily reduce many clinical difficulties. In the context, the current study conducted to evaluate the psychological well-being of substance use patients in comparison of combined religious therapy and pharmacotherapy therapy. The result indicated that religious therapy plays role in increasing the psychological well-being of drug addicts. Moreover, the means difference in the pre and posttest of experimental group indicated a difference thus, confirming that religious therapy had a role in handling or boosting the psychological well-being of drug addicts. The findings are consistent with the previous researches that high religious participation was linked with a reduced probability of alcohol or drug use. The association between religious activities and prevention or modest alcohol use is well acknowledged, whether or not denominational tenets specifically prohibit the use of alcohol.^21^ Further, a review of literature confirmed the role of spiritualty in dealing with the problem of addiction.^22^ Religious adolescents in particular have been found to be less likely to use alcohol or marijuana in relation to their less irreligious counterparts.^23^ It has been witnessed that many religions have formalized addiction handling programs. The Jewish, Catholic and Muslim offer religious therapy to the substance use patients with the problems of depression and suicides. The use of religion put person focus on the God to overcome their problem and get extra strength by meeting people of their same problem. If religious leaders encourage the substance use victims, it can be beneficial for many.^24^

Among the religious practices, there might be many factors that contributed to this result. The drug addicts suffered from feeling of isolation, low confidence, poor decision making ability and received ignorance from close ones. In these problems religion helps the people to acquire self-confidence, becomes aware about their abilities and skills provide help to turn down the distresses and problems. Further, the religion also discourages the drug use and brings a healthy transformation in the victim which is helpful in the treatment of drug addiction. A review of the literature gives indications that religious conviction considerably affects people’s happiness and well-being. Further, an increase in religious practice inculcate high level of hope and an enhanced sense of purposeful life.^25^ Finally another study, proved that meditative prayer is related to positive feelings of existential well-being and satisfaction related to religion. Further, it provides the information on intrinsic religious orientation and church activity which proved the fact that the passionate religious practices such as religious mysticism occur in the lives of the religious devout and are not restricted to the church attendees.^26^

We also assessed the psychological well-being of drug addicts in comparison with demographics characteristics. The results on role of age groups on psychological well-being indicated that there was not a remarkable difference in the means of pre and posttest of different age groups. Research has shown that an effective therapist pays importance to the age characteristics of the person while improvising therapy^27^, while on the other hand the current research has showed that age levels do not hinder the effectiveness of religious therapy in improving the psychological well-being of drug addicts.

There was not a notable difference in the means of posttest of marital status of the patients in our study. It means religious activities have a positive effect in enhancing the psychological well-being of drug addicts on their marital status. The marital status of drug addicts has shown no different effect on enhancing psychological well-being but research confirmed that marital status effects the outcome of cognitive behavior therapy for marijuana addicts.^23^ beside other studies have also confirmed that separated person give more promising substance outcomes as comparing to married or single.^28^

It can be concluded that religion is equally important for all educational levels whereas, in contrast to current study findings, previous research concluded that the literate individuals give more arguments, are less doubtful and more protective to the therapy. There was a weak positive correlation found between education and the religious participation indicating that high education may trigger individual towards religion.^29^

The variable of residential type indicated that rural area had high mean as compare to urban area and a study conducted on rural older adults confirmed that these participants were more prone to consult the mental health specialists if referred by any physician.^30^

Finally, high income patients show better psychological well-being compared with to the low income. The research also identified that therapy might be more fruitful to high income individuals as compared to low income. The assumption may be justified as high income persons do not face any economic pressure whereas people with less income suffered a lot of pressure, depression and dissatisfaction to their lifes.^31^

## CONCLUSION

Religious therapy had a significant therapeutic effects on psychological well-being of the substance use patients and it is equally effective for all age group, marital status and educational level.

### Author`s Contribution

**ZB and IN** conceived, designed and did statistical analysis & editing of manuscript.

**NL and ZB** did data collection and manuscript writing.

**IA** did review and final approval of manuscript.
